# Natural Deep Eutectic Solvents (NADES) in Food Systems: Emerging Applications, Extraction Efficiency, Safety Concerns, and Regulatory Challenges

**DOI:** 10.1021/acs.jafc.5c11656

**Published:** 2025-12-18

**Authors:** Karina Pinheiro Martins, Nathália Letícia Hernandez Brito, Gustavo Barreto Costa, Jhony Silva Ramos, Ana Caroline Silvestre Barbosa Alessi, Thais Aparecida Santos, Ana Elisa Melo da Silva, Isaac Felipe Machado, Leila Larisa Medeiros Marques, Adriana Aparecida Droval Arcain, Flávia Aparecida Reitz Cardoso

**Affiliations:** † Postgraduate Program of Food Technology (PPGTA), 74354 Federal University of Technology - Paraná (UTFPR), Campo Mourão, Paraná 87301-005, Brazil; ‡ Postgraduate Program of Technological Innovations (PPGIT), Federal University of Technology - Paraná, Campo Mourão, Paraná 87301-005, Brazil; § Department of Food Engineering and Chemical Engineering, Federal University of Technology - Paraná (UTFPR), Campo Mourão, Paraná 87301-005, Brazil

**Keywords:** natural deep eutectic solvents, green extraction, food applications, bioactive compounds, food safety, sustainable technologies

## Abstract

Natural deep eutectic solvents (NADES) have emerged as green alternatives to conventional organic solvents in food processing. They are biodegradable, nonvolatile, and highly efficient at solubilizing bioactive molecules, including polyphenols, flavonoids, anthocyanins, carotenoids, and alkaloids. From 2020 to 2025, significant advances have clarified their composition, physicochemical behavior, interaction mechanisms, and technological uses in foods. Rational combinations of hydrogen-bond donors and acceptors, often composed of substances classified as Generally Recognized as Safe (GRAS), enable selective and high-yield extraction from plants, agro-industrial byproducts, and food waste. Coupling NADES with ultrasound, microwave, or pressurized extraction has increased efficiency while protecting heat-sensitive compounds. Beyond extraction, NADES show promise as preservatives, delivery systems, encapsulating media, color stabilizers, and natural antioxidants in meat, dairy, fermented, and plant-based products. Remaining bottlenecks include high viscosity, scarce toxicological and metabolic data, absence of specific regulations, and limited research on sensory impacts and shelf life under realistic large-scale industrial conditions.

## Graphical abstract

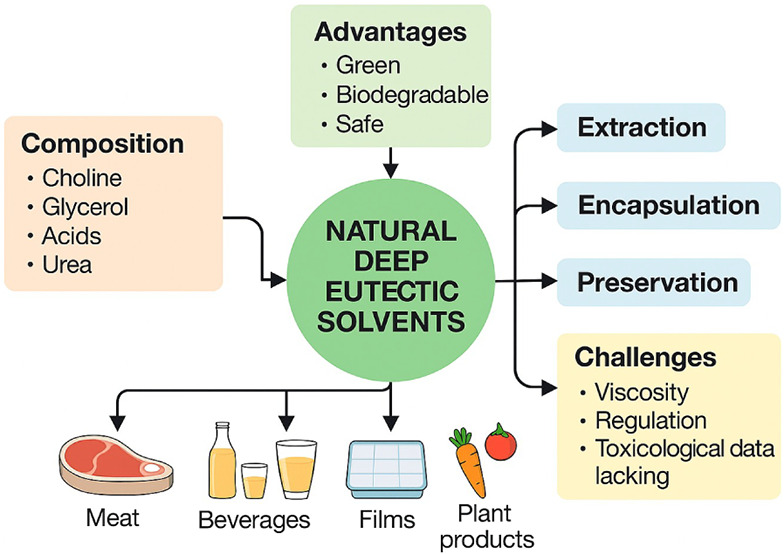

## 1 Introduction

The global pursuit of healthier, more sustainable, and functional foods has intensified the search for innovative solvents and processing technologies that can efficiently extract and stabilize bioactive compounds.^1,2^ Natural deep eutectic solvents (NADES) have emerged as one of the most promising alternatives to volatile organic solvents, combining environmental safety, biodegradability, and high extraction selectivity.^3,4^

Historically, the concept of deep eutectic solvents was first established by Dai et al.,^5^ who demonstrated that eutectic mixtures based on choline chloride and carboxylic acids could act as efficient, tunable solvents. Paiva et al.^6^ and Dai et al.^7^ later introduced the concept of natural deep eutectic solvents, showing that mixtures of sugars, amino acids, and organic acids could form stable liquids with low vapor pressure and broad solvent capacity. These pioneering studies established the molecular basis for NADES design and remain essential to understanding their physicochemical behavior.

Unlike synthetic ionic liquids, NADES can be entirely composed of substances classified as Generally Recognized as Safe (GRAS) by the FDA, making them highly attractive for direct use in food matrices without requiring solvent removal.^8^ Their ability to modulate polarity and stabilize phenolic compounds, flavonoids, carotenoids, and alkaloids is primarily attributed to dense hydrogen-bond networks, which reduce oxidative degradation and promote selective solubilization.^2,9^

Over the past decade, the field has evolved from proof-of-concept studies to application-driven investigations integrating NADES into green extraction and food formulation systems. However, despite an exponential increase in publications since 2020, the literature still lacks comparative analyses, systematic evaluation of unsuccessful applications, and comprehensive toxicological or sensory data. This review aims to bridge these gaps by offering a critical synthesis that integrates mechanistic understanding, recent technological advances, and regulatory perspectives.

NADES are liquid systems formed by two or more natural substances (such as amino acids, sugars, alcohols, organic acids, or nitrogenous bases) which, when mixed in specific proportions, exhibit a melting point lower than that of their components.^8,15^ This property allows the formation of highly viscous systems with strong hydrogen bonding interactions, making them capable of extracting, protecting, and stabilizing phenolic compounds, flavonoids, carotenoids, and alkaloids, among others.^2,7^

The primary advantage of NADES over volatile organic solvents such as methanol, ethanol, and acetone lies in their natural origin and food-grade safety. They can be formulated using components classified as GRAS by the FDA.^8^ This characteristic makes NADES particularly appealing for applications in the food industry, as they significantly reduce toxicological risks and do not leave harmful residues for either human health or the environment.^9,10^

In recent decades, scientific interest in NADES has grown significantly, with most studies focusing on their efficiency in extracting bioactive compounds, such as polyphenols, anthocyanins, saponins, and carotenoids, from various plant matrices and agro-industrial byproducts.^1,11^ Recent findings indicate that NADES can substantially enhance both the yield and stability of antioxidant compounds, thereby increasing their applicability in functional formulations, including beverages, meat products, and baked goods.^12,13^

Moreover, the ability of NADES to modulate the polarity of the extraction system, their tunable selectivity, and compatibility with green technologies—such as ultrasound-assisted extraction (UAE), microwave-assisted extraction (MAE), and pressurized liquid extraction (PLE)—underscore their versatility in sustainable bioprocessing.^10,14^ The combination of these techniques with NADES has led to significant improvements in extraction time, energy efficiency, and compound selectivity, positioning NADES as a viable and eco-friendly alternative to conventional solvents.

Table 1 presents a comparative overview of major green extraction techniques combined with NADES, highlighting commonly used systems, average yield improvements, and operational limitations.

**Table 1:** Extraction Techniques Coupled with NADES: Examples, Yield Improvements, and Limitations (2020–2025)

Technique	Example of NADES system	Average yield increase (%)	Advantages	Limitations	References
Ultrasound-Assisted Extraction (UAE)	choline:glycerol	35%	Reduces extraction time and improves NADES penetration	Requires optimization of sonication power and time	Jelić et al.;^3^ Pal and Jadeja^11^
Microwave-Assisted Extraction (MAE)	choline:fructose	40%	Fast heating and intensified mass transfer with NADES	Risk of compound degradation under high-energy conditions	Molnar et al.;^2^ Ran et al.^1^
Pressurized Liquid Extraction (PLE)	betaine:citric acid	50%	High efficiency under moderate temperature and pressure	Expensive equipment and the need for pressure-resistant vessels	Radošević et al.;^10^ Abbott et al.^4^
Conventional solvent extraction	ethanol:water	—	Widely used and easily accessible	Environmental and toxicological issues of organic solvents	Molnar et al.;^2^ Ran et al.^1^

However, despite recent progress, the use of NADES in food applications still faces significant challenges.

Key limitations include the lack of systematic toxicological studies, insufficient assessment of their interactions with food matrices, and the absence of specific regulations authorizing their use as solvents or technological coadjuvants in food products.^1,2^ In addition, many studies remain restricted to laboratory-scale conditions, often neglecting critical aspects such as sensory evaluation, shelf life, industrial scalability, and the long-term stability of NADES-based extracts.^13^

It is particularly important to note that, unlike traditional solvents, NADES are not typically removed from the final extract in most applications. This characteristic demands further investigation into their direct use in food systems, including studies on digestibility, metabolic behavior, effects on the gut microbiota, and the potential impact on the nutritional profile of final products.^1,9^ Some studies suggest that NADES may function as bioactive carriers, protecting sensitive compounds from thermal and oxidative degradation. However, their interactions within complex food matrices remain insufficiently understood and require deeper exploration.

Another limitation observed in the current literature is the disproportionate focus on a narrow range of NADES formulations, especially those based on choline:glycerol or choline:lactic acid, leaving a wide variety of potential combinations underexplored. Research involving smart NADES responsive to environmental stimuli (e.g., pH, enzymes, temperature) remains limited, as researchers focus on multicomponent systems and advanced applications, such as controlled-release or encapsulation strategies.^2,14^ The development of customized NADES tailored to specific compounds and food applications thus represents a promising but largely unexplored frontier.

In this context, the present article aims to provide a critical, comprehensive, and up-to-date review (2020–2025) of natural deep eutectic solvents in food systems, with a particular focus on:

1. The composition, physicochemical properties, and variability of NADES formulations.
2. Extraction techniques combined with NADES and their technological advantages.
3. Applications across different food matrices and categories of bioactive compounds.
4. Current limitations, regulatory challenges, and future perspectives for industrial-scale use.

This review consolidates recent scientific advancements and includes case studies involving NADES applications in both plant- and animal-based food systems, such as *Talisia esculenta* and *Stachys byzantina*. Additionally, it broadens the scope to encompass emerging applications in fermented foods, clean-label formulations, and plant-based products. The article also offers a critical analysis of the toxicological, regulatory, and technological barriers that must be addressed to enable the broader adoption of NADES in the food industry.

## 2 Composition and Properties of Natural Deep Eutectic Solvents (NADES)

The structural and functional behavior of NADES depends primarily on the physicochemical characteristics of their constituent components. Understanding their composition, molecular interactions, and tunable properties is fundamental for optimizing their performance in food systems. This section describes the chemical principles that govern the formation of NADES, the types of hydrogen bond donors (HBDs) and acceptors (HBAs) typically used, and how their physicochemical parameters, such as viscosity, polarity, and stability, affect extraction efficiency and functionality in food applications.

### 2.1 Concept and Formation Mechanisms

Natural Deep Eutectic Solvents (NADES) are liquid systems formed by combining hydrogen-bond donors (HBDs) and acceptors (HBAs), whose supramolecular interactions disrupt the crystalline lattice of each component and result in the formation of a stable, homogeneous liquid at room temperature.^7,16^ These interactions, primarily hydrogen bonding, van der Waals forces, and dipole–dipole attractions, result in a significant decrease in the system’s melting point compared to the pure constituents, thereby lowering its free energy and enhancing thermodynamic stability.^9^

The structural reorganization of the components within NADES produces highly viscous, nonvolatile, and thermally stable liquids with tunable polarity, properties that distinguish them from conventional organic solvents. Because these features can be tailored by adjusting the type and molar ratio of HBA and HBD, NADES are now recognized as versatile and sustainable media suitable for applications in food processing, pharmaceuticals, and cosmetics.^2^

### 2.2 Types of HBA and HBD and Their Selection

The selection of components is a critical factor in optimizing the application of NADES. The most commonly used hydrogen bond acceptors (HBAs) include choline chloride, betaine, l-proline, ethanolamine, and various quaternary ammonium derivatives.^17^ Hydrogen bond donors (HBDs) can be categorized into organic acids (e.g., lactic, citric, and malic acids), polyols (e.g., glycerol and sorbitol), urea, and mono- or disaccharides, such as glucose and fructose.

For food-related applications, the selected components must be recognized as generally recognized as safe (GRAS) by the FDA, thereby minimizing toxicological risks. Recent studies have demonstrated that a 1:2 molar ratio of choline and glycerol is particularly effective in extracting polyphenols from plant matrices, including those from red onion and tomato residues.^11^

In addition, the selection of HBA:HBD pairs must be tailored to the polarity of the target compounds. Acidic NADES formulations are more suitable for extracting ionizable phenolic compounds, whereas systems based on polyols or urea are generally more effective for flavonoids and alkaloids.^1^

As summarized in Table 2, different HBA:HBD combinations yield solvents with distinct physicochemical properties and application-specific functionalities. For instance, the choline:glycerol (1:2) system is widely used for polyphenol extraction due to its high polarity. At the same time, a menthol:caprylic acid mixture is more appropriate for the recovery of lipophilic compounds. These variations highlight the importance of carefully selecting eutectic pairs based on the chemical nature of each bioactive target.^1,8^

**Table 2:** Typical NADES Compositions Used in Food Applications

HBA	HBD	Molar ratio	Typical application	Reference
Choline chloride	Glycerol	1:2	Polyphenol extraction from residues	Janić et al.^18^
Choline chloride	Lactic acid	1:1	Phenolic compounds from grape skins	Vanda et al.^19^
Choline chloride	Citric acid	1:1	Flavonoid extraction from onions	Smith et al.^20^
Betaine	Fructose	1:1	Stabilization of antioxidant compounds	Aslan and Doğan^17^
Proline	Malic acid	1:2	Pigment extraction from vegetables	Pal and Jadeja^11^

The interplay between molecular composition, molar ratio, and water content, therefore, governs the physicochemical behavior of NADES. Understanding how these variables interact is essential for predicting viscosity, polarity, and extraction selectivity, which are discussed in the following subsections.

### 2.3 Physicochemical Properties of NADES

#### 2.3.1 Viscosity

High viscosity is among the main operational limitations of NADES. It varies widely (100–10,000 mPa·s) depending on the molecular structure and hydrogen-bond network density of the components. Generally, increasing the proportion of polyol-type hydrogen bond donors (HBDs), such as glycerol or sorbitol, leads to stronger intermolecular associations and, consequently, higher viscosity. Conversely, higher water content or the use of smaller organic acids decreases viscosity due to disruption of the hydrogen-bond lattice.^1^

For example, in choline-based systems, changing the molar ratio from 1:1 to 1:3 with glycerol increases viscosity from approximately 400 to 1200 mPa·s. In contrast, the addition of 30% water can reduce it to 270 mPa·s without compromising extraction efficiency.^11^ This demonstrates that viscosity is not only composition-dependent but also modulated by hydration and temperature, both of which alter the supramolecular organization of the solvent.

As shown in Table 3, the viscosity and polarity of NADES vary widely according to component type and water content, which explains the differences in extraction efficiency reported for each bioactive class.

**Table 3:** Characteristics of Different NADES Systems Applied to the Extraction of Bioactive Compounds (2020–2025)

HBA	HBD	Molar ratio	Polarity	Viscosity (mPa·s)	Target application	Reference
Choline	Glycerol	1:2	Hydrophilic	1200 (without water)	Polyphenols from red onion	Pal and Jadeja^11^
Choline	Lactic acid	1:2	Hydrophilic	850 (with 20% water)	Flavonoids from grape seed	Ran et al.^1^
Choline	Urea	1:2	Hydrophilic	420	Alkaloids from green tea leaves	Zhou et al.^21^
Choline	Citric acid	1:1	Hydrophilic	980	Bioactive compounds from *Talisia esculenta*	Souza et al.^22^
Betaine	Malic acid	1:2	Hydrophilic	700 (with water)	Phenolic compounds from olive	Molnar et al.^2^
Menthol	Caprylic acid	1:1	Hydrophobic	180	Carotenoids from carrot and bell pepper	Radošević et al.^10^
Choline	Acetic acid	1:1	Hydrophilic	860 (heated)	Phenolic compounds from *Stachys byzantina*	Da Silva et al.^23^
Choline	Fructose	1:1	Moderate	1500 (without water)	Anthocyanin extraction from red grapes	Aslan and Doğan^17^
Choline	Sorbitol	1:2	Hydrophilic	980	Polyphenols from tomato residues	Zainal-Abidin et al.^8^

Adding water is a common strategy to reduce viscosity and improve molecular diffusion. Pal and Jadeja^11^ reported a 77% reduction in the viscosity of choline:glycerol (1:2) with the addition of 30% water, without compromising extraction efficiency.

#### 2.3.2 Polarity

Polarity is one of the most critical physicochemical parameters influencing both solubility and extraction selectivity in NADES. Hydrophilic systems, such as choline:glycerol, choline:lactic acid, and betaine:citric acid, exhibit high dielectric constants (ε ≈ 55–65), indicating superior performance for highly polar antioxidants, including phenolic acids and anthocyanins. In contrast, hydrophobic NADES, such as menthol:caprylic acid or dodecanol:lauric acid, display dielectric constants below 15 and are more efficient for carotenoids, chlorophylls, and lipophilic tocopherols.^10^

Unlike conventional binary systems, such as ethanol–water, whose polarity range is narrow, the polarity of NADES can be precisely tuned by adjusting the component type and ratio. For instance, increasing the acid content in choline:lactic acid systems enhances polarity and proton-donor capacity, improving the extraction of quercetin and rutin.^2^ Conversely, substituting glycerol with sugars like fructose increases the number of hydrogen-bonding sites but decreases dielectric strength, favoring moderately polar molecules such as flavan-3-ols.^17^

Comparative analyses indicate that polarity alone does not determine extraction efficiency; viscosity and molecular-size compatibility also play decisive roles. Ran et al.^1^ observed that despite similar dielectric constants, choline:lactic acid (1:2) outperformed choline:glycerol (1:2) for flavonoid extraction due to its lower viscosity and higher diffusivity. This highlights the need for integrated evaluation of polarity-viscosity relationships rather than isolated parameter analysis.

Overall, the tunable polarity of NADES offers a mechanistic advantage over traditional solvents, enabling the selective recovery of diverse bioactive classes within a single sustainable framework.

#### 2.3.3 pH, Stability, and Conductivity

Water acts as a critical physicochemical modulator in NADES, altering viscosity, polarity, and conductivity. While small amounts of water (10–30%) enhance mass transfer and diffusion, excessive dilution (>50%) can disrupt the eutectic network, transforming the mixture into a conventional aqueous solution.^17^ Therefore, controlled water addition is essential to maintain the cooperative hydrogen-bond interactions that are responsible for the stability and solvent properties of NADES.

From an extraction perspective, water not only decreases viscosity but also affects the solvation of phenolic compounds by altering dipole–dipole interactions and proton-donor capacity. The optimum balance typically lies between 20% and 40% water, which preserves structure while improving kinetics. This tunability underscores the potential of NADES as customizable solvents for both hydrophilic and amphiphilic molecules.

The physicochemical parameters of NADES are interdependent, as viscosity, polarity, and conductivity vary simultaneously with changes in composition and hydration. Mastering these relationships allows rational solvent design for specific extraction purposes, as further illustrated in Section 2.4.

### 2.4 Influence of Molar Ratio and Water Addition

The molar ratio between components directly affects the system’s viscosity, stability, and extraction efficiency. A 1:1 ratio tends to result in higher viscosity, while 1:2 and 1:3 ratios promote greater molecular mobility. Ran et al.^1^ reported a 37% increase in flavonoid extraction using choline:lactic acid (1:2) with 20% water.

Figure 1 illustrates the progressive reduction in viscosity of the choline:glycerol (1:2) mixture with increasing water content. A significant drop is observed from 1200 mPa·s (0%) to 270 mPa·s (30%), which confirms the findings of Pal and Jadeja,^11^ and reinforces the strategic role of water as a physicochemical modulator. This trend aligns with several studies indicating that additions between 20% and 40% preserve intermolecular bonding integrity without compromising extraction potential (Aslan and Doğan^17^).

**Figure 1:**
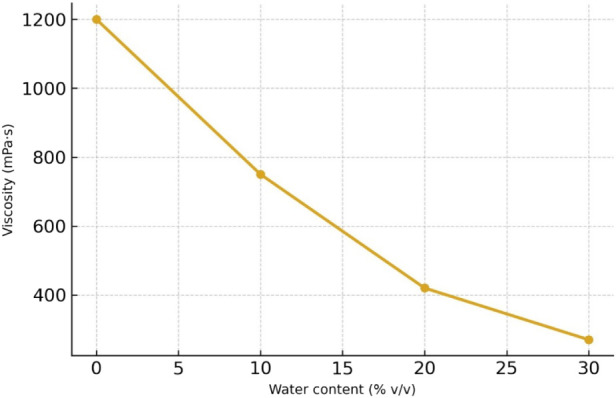
Effect of water addition on viscosity of choline:glycerol NADES (1:2).

### 2.5 Functional Classification and Application

NADES can be functionally categorized into three complementary classes, each defined by its operational mechanism within the food processing workflow:

1. Extraction-oriented NADES, which serve as direct solvents for recovering phenolic compounds, carotenoids, alkaloids, and saponins from natural matrices. Their performance depends on polarity, viscosity, and the presence of stabilizing hydrogen bonds.
2. Pretreatment NADES, applied to soften plant cell walls, disrupt lignocellulosic structures, or inactivate oxidative enzymes before extraction, thereby enhancing yield and purity.
3. Carrier or encapsulating NADES, which function as in situ stabilizers and delivery systems, forming supramolecular cages around sensitive molecules. These systems show promise in protecting anthocyanins, catechins, and vitamins from thermal or oxidative stress.^2^

This classification provides a mechanistic basis for tailoring NADES design to specific process stages, whether to enhance solubilization, facilitate extraction, or ensure bioactive stability. Each functional class presents distinct advantages and constraints. Extraction-oriented NADES maximize yield but often require postprocessing purification due to high viscosity; pretreatment NADES improve cell-wall disruption yet may alter enzymatic activity or pigment integrity; and carrier NADES enhance stability and bioavailability but raise concerns regarding sensory neutrality and metabolization. Few comparative studies systematically quantify these trade-offs, and most literature emphasizes extraction efficiency over formulation compatibility. Addressing these gaps will be essential to guide industrial applications. In practice, this functional division also enables comparative evaluation between systems optimized for process efficiency and those intended to act as active components in food formulations, a distinction that remains insufficiently emphasized in most recent reviews.

Molnar et al.^2^ reported that NADES formulated with betaine and dicarboxylic acids are particularly suitable for encapsulating unstable compounds such as anthocyanins and catechins, due to the strong hydrogen-bond networks that minimize molecular oxidation and thermal degradation. Similarly, betaine:malic acid systems demonstrated superior retention of catechins during simulated digestion compared to ethanol controls, suggesting the potential for NADES to enhance the physiological stability of polyphenolic compounds.

In a recent study, Souza et al.^22^ demonstrated that citric acid–based NADES effectively extracted bioactive compounds from *Talisia esculenta*, and the resulting extracts were successfully incorporated into fresh sausages, enhancing oxidative stability and sensory acceptability. Extracts of *Stachys byzantina* obtained using a choline:acetic acid (1:1) ratio showed a high recovery of antioxidant compounds.^23^ Their application in meat products contributed to improved lipid and color stability during storage.^12^ These examples illustrate the translational potential of NADES-derived extracts, confirming their capacity to progress from laboratory-scale studies to functional food applications with measurable sensory and microbiological benefits.

Taken together, the available evidence demonstrates that NADES perform multiple roles across food processing systems, ranging from efficient extraction and stabilization to encapsulation and direct incorporation. This multifunctionality distinguishes NADES from conventional solvents by combining extractive capacity with bioactive protection, thereby supporting their inclusion in integrated biorefinery and circular economy frameworks.

### 2.6 Limitations and Gaps

Despite significant progress, the large-scale application of NADES still faces several important limitations:

(i) high viscosity, which impairs mass transfer and hinders their use in continuous industrial processes;
(ii) absence of specific regulatory frameworks from agencies such as EFSA and FDA;
(iii) limited availability of toxicological and biodegradability studies;
(iv) challenges related to the efficient recovery and reuse of the solvent.

According to Suresh et al.,^9^ only 18% of studies published between 2020 and 2024 report experimental data on NADES reuse, and fewer than 10% include sensory impact assessments of the final product. This gap is particularly critical for food industry applications, where consumer acceptance is strongly influenced by the product’s sensory attributes.

Additionally, most existing research is limited to simplified laboratory models, typically involving dried leaves or powdered plant materials, with limited extrapolation to more complex food systems, such as emulsions, meat products, or fermented beverages. The relationship between the molecular structure of NADES and their ability to stabilize bioactive compounds remains poorly understood and underexplored.

Industrial-scale implementation also requires addressing several practical constraints, including:

- Cost optimization, as the price of GRAS-certified reagents remains relatively high.
- Standardization of synthesis routes and quality control parameters.
- Comprehensive risk assessments and food safety evaluations.

In this context, advancing the application of NADES in food systems will depend on a combination of strategies, including the rational selection of HBA:HBD pairs based on target compound characteristics, viscosity modulation through the incorporation of water or cosolvents, and alignment with regulatory standards and sensory quality requirements.

The physicochemical tunability of NADES provides a versatile platform for tailoring extraction selectivity; however, the limited mechanistic understanding of hydrogen-bond dynamics under real processing conditions still represents a major research gap.

While NADES exhibit remarkable tunability and sustainability advantages, their widespread use in the food industry depends on bridging the gap between laboratory optimization and real-scale processing. Future research must integrate molecular modeling, process engineering, and toxicological validation to transform NADES from an academic concept into a fully deployable green-solvent platform.

## 3 Applications of NADES in the Food Industry

The use of natural deep eutectic solvents (NADES) in the food industry has grown as a sustainable alternative to conventional organic solvents such as ethanol, methanol, and acetone. Their ability to solubilize bioactive compounds, with low toxicity, food compatibility, and ease of preparation, makes NADES particularly attractive for applications involving the extraction, stabilization, and incorporation of functional ingredients. This section discusses the main applications of NADES in foods, organized by bioactive compound group and emerging technology. However, most studies have emphasized extraction yield rather than the physicochemical or sensory implications of incorporating NADES into foods, which limits our understanding of their broader functional potential.

### 3.1 Phenolic Compounds and Flavonoids

Phenolic compounds are the most extensively studied phytochemicals in combination with NADES due to their antioxidant and antimicrobial properties, as well as their potential as natural preservatives. Several hydrophilic NADES have performed superior to conventional solvents in extracting these compounds.

Recent studies using choline chloride-based NADES for onion byproducts have shown that these systems can significantly enhance the recovery of phenolic antioxidants compared to conventional hydroalcoholic solvents.^11^ Ran et al.^1^ reported optimized flavonoid extraction from grape seeds using choline:lactic acid (1:2), maintaining 92% of antioxidant activity after 15 days of storage. Souza et al.^22^ reported that choline:citric acid NADES (1:1) effectively extracted phenolics from *Talisia esculenta*, which were successfully applied in fresh sausages, improving oxidative and sensory stability. Ran et al.^1^ reported optimized flavonoid extraction from grape seeds using choline:lactic acid (1:2), maintaining 92% of antioxidant activity after 15 days of storage. Souza et al.^22^ reported that choline:citric acid NADES (1:1) effectively extracted phenolics from *Talisia esculenta*, which were successfully applied in fresh sausages, improving oxidative and sensory stability.

These studies consistently demonstrate that NADES composed of choline or organic acids outperform ethanol-based systems in terms of selectivity and stability. Nevertheless, comparative studies using identical matrices and extraction conditions are still lacking, preventing a clear understanding of how molar ratio, viscosity, and hydrogen-bond donor structure govern the solubilization mechanism. Moreover, negative or neutral findings remain largely unreported. Bi et al.,^24^ for instance, observed that high-viscosity sugar-based NADES reduced phenolic recovery to less than 60% of that achieved with ethanol. Such contradictory evidence highlights the need for standardized protocols and quantitative benchmarks to separate the true efficiency of NADES from matrix-dependent variability.

### 3.2 Carotenoids and Lipophilic Compounds

Carotenoids and other lipophilic compounds represent a major challenge in food extraction due to their low solubility in polar solvents and high sensitivity to oxidation, light, and temperature. Hydrophobic and amphiphilic NADES have emerged as promising alternatives to conventional organic solvents such as hexane and acetone, offering higher safety, tunability, and environmental compatibility.

Radošević et al.^10^ demonstrated that a menthol:caprylic acid (1:1) NADES increased the recovery of β-carotene from carrot residues by 34% compared with hexane, while maintaining superior antioxidant capacity. Jelić et al.^3^ successfully applied a dodecanol:lauric acid NADES for the extraction of astaxanthin from microalgae, reporting a 25% higher yield and improved color stability during storage.

These results indicate that the hydrogen-bonding and van der Waals interactions in hydrophobic NADES provide a stabilizing microenvironment, protecting unsaturated structures from isomerization and oxidative degradation.

In addition to extraction efficiency, the physicochemical properties of NADES, particularly viscosity, polarity, and water content, strongly influence carotenoid solubility and stability. Increasing the water fraction to 30% reduces viscosity and facilitates diffusion without disrupting the eutectic network, resulting in higher mass transfer rates and improved pigment recovery. Conversely, excessive dilution (>50%) can collapse the hydrogen-bond network, decreasing selectivity and pigment retention.^17^

Recent studies have also explored the use of hydrophobic NADES for direct incorporation of carotenoid-rich extracts into food matrices. For instance, de Andrade et al.^25^ formulated β-carotene-enriched oil emulsions stabilized with menthol-based NADES, achieving enhanced oxidative stability and uniform pigment dispersion. Such strategies align with clean-label trends, as the NADES themselves act as both solvents and stabilizing agents, eliminating the need for toxic residues or additional emulsifiers.

Despite these promising advances, certain limitations persist. Hydrophobic NADES often exhibit high viscosity, which can hinder homogeneous dispersion in aqueous or emulsion-based foods. Furthermore, phase separation and incomplete solubilization of carotenoids in complex matrices remain unsolved issues.

Comparative studies on mass transfer kinetics, solvation thermodynamics, and reusability of these systems are scarce, and most works neglect to evaluate color stability or antioxidant retention during shelf life.

Therefore, a critical research priority is to develop amphiphilic NADES combining hydrophilic and lipophilic components to achieve an optimal balance between extraction efficiency and food compatibility. Mechanistic studies focusing on pigment–NADES interactions (π–π stacking, dipole orientation, and hydrogen-bonding dynamics) are also required to clarify the molecular basis of stabilization and guide rational solvent design.

### 3.3 Anthocyanins and Natural Pigments

Anthocyanins are among the most unstable classes of natural pigments, being highly sensitive to temperature fluctuations, pH changes, and exposure to light. These molecules contribute to the visual and antioxidant properties of beverages, fruit-based products, and functional foods. NADES have demonstrated an exceptional ability to both extract and stabilize anthocyanins, primarily due to their strong hydrogen-bonding properties and the ability to create microenvironments with reduced water activity.^2,17^

Aslan and Doğan^17^ demonstrated that choline:fructose (1:1) preserved 88% of anthocyanin color intensity after 7 days at 25 °C, compared with only 63% retention using ethanol. This stabilization effect results from the formation of hydrogen bonds between the hydroxyl groups of anthocyanins and the sugar-based NADES network, which reduces molecular mobility and prevents hydration of the flavylium cation.

Similarly, Radošević et al.^10^ reported that NADES, composed of glucose and citric acid, extended anthocyanin stability by 40% at neutral pH, enabling applications in dairy and plant-based matrices. This ability to modulate the polarity and pH microenvironment of NADES makes them highly adaptable for pigment stabilization in complex food systems.

However, research gaps remain significant. Most studies measure color retention under controlled conditions but lack kinetic modeling of degradation reactions, including first-order constants and activation energy for thermal decomposition. Furthermore, few works address pigment–protein or pigment–polysaccharide interactions, which are critical for real food matrices.

Future studies integrating spectroscopic and thermodynamic analyses could elucidate copigmentation mechanisms and optimize NADES formulations for chromatic stability during processing and storage.

### 3.4 Alkaloids, Bitter Compounds, and Saponins

Alkaloids, caffeine, theobromine, and saponins play dual roles in foods, providing bioactivity while also contributing to the bitterness and astringency of these compounds. NADES have demonstrated strong selectivity for extracting these nitrogen-containing compounds, offering safer and more environmentally friendly alternatives to methanol and chloroform.

Zhou et al.^21^ achieved 94% caffeine recovery from green coffee husks using a choline:urea (1:2) NADES, outperforming ethanol and ethyl acetate while reducing solvent toxicity. Taco et al.^13^ observed that a choline:glycerol (1:3) ratio selectively extracted saponins from quinoa husks, reducing residual bitterness by 38% and improving sensory quality in reconstituted beverages.

The efficiency of NADES in alkaloid extraction arises from the proton-donor and acceptor capacity of their functional groups, which facilitate ionic and π–π interactions with heterocyclic alkaloids. This allows higher selectivity and recovery even at mild temperatures.

Nevertheless, several limitations persist. The basicity of some NADES components can lead to partial degradation of labile alkaloids, while residual solvent taste has been reported in caffeine-rich extracts when purification steps are omitted.^11^ More systematic studies on solvent–solute equilibria, p*K*_a_ shifts, and protonation dynamics are required to predict extraction behavior and avoid undesired chemical transformations.

From an application perspective, alkaloid and saponin extracts obtained via NADES could be used to modulate flavor and functionality in cocoa-based products, teas, and nutraceuticals. However, quantitative correlations between extraction parameters and sensory outcomes remain scarce, representing an essential area for future development.

### 3.5 Direct Application of NADES in Foods

Beyond their role as extractants, food-grade NADES are being increasingly explored for direct incorporation into food matrices, where they can serve as multifunctional components, acting as solvents, stabilizers, or preservatives. Although many of their individual components are classified as GRAS, the eutectic mixtures themselves are not automatically GRAS; consequently, the safety of each specific NADES must be experimentally assessed before allowing its direct presence in the final product, especially in food applications.^1,2,22^

Souza et al.^22^ incorporated citric acid–based NADES extracts from *Talisia esculenta* directly into fresh sausages, observing lower lipid oxidation, reduced microbial growth, and preserved sensory acceptability over a 10-day storage period. Silva et al.^12^ reported comparable outcomes using choline:acetic acid (1:1) extracts of *Stachys byzantina* in pork sausages, noting improved color stability and antioxidant capacity during refrigerated storage.

These studies illustrate a promising shift from extraction-focused to application-oriented research on NADES. In such systems, NADES not only extract bioactives but also act as carriers, enhancing solubility and stability within the food matrix.

However, a comprehensive evaluation of the metabolic fate of NADES in the human body remains lacking. Studies on gastrointestinal digestion and absorption kinetics are necessary to ensure the long-term safety of these products.

Another critical gap lies in sensory evaluation. Although preliminary data indicate minimal impact on flavor and aroma, no standardized protocols exist to assess thresholds of NADES detection or potential masking effects on volatile compounds.

Consumer perception and descriptive sensory analyses (triangle tests, preference mapping) should be incorporated into future research to validate the acceptance of NADES-containing foods.

From a technological perspective, the rheological and physicochemical behavior of NADES in heterogeneous matrices, such as doughs, emulsions, and fermented products, must also be clarified, as these parameters influence both processability and bioactive stability. Exploring matrix–solvent interactions (protein denaturation, lipid partitioning, and pH buffering) could provide critical insights for industrial-scale applications.

### 3.6 Encapsulation and Controlled Release

Several NADES have demonstrated the ability to protect unstable compounds such as vitamin C, flavonoids, and probiotics. Molnar et al.^2^ used betaine:malic acid (1:2) NADES to protect catechins during simulated digestion. Suresh et al.^9^ observed microdomains that enable prolonged intestinal release, suggesting potential applications in targeted functional foods.

These systems are compatible with emulsions, enteric capsules, and microencapsulated foods; however, industrial-scale validation is still required.

Encapsulation and controlled release of bioactive compounds have emerged as strategic approaches to enhance stability, improve bioavailability, and facilitate targeted delivery in functional foods. NADES offer a unique platform for these applications due to their supramolecular structure and tunable polarity, which allows stabilization of sensitive compounds and modulation of release profiles.^2,9^

Molnar et al.^2^ demonstrated that betaine:malic acid (1:2) effectively protected catechins during simulated gastrointestinal digestion, retaining 92% of total antioxidant capacity compared with 60% in control emulsions. Similarly, Suresh et al.^9^ reported that glycerol-based NADES facilitated the gradual release of encapsulated phenolic compounds during intestinal digestion, increasing their bioaccessibility and reducing oxidative degradation.

NADES can be combined with biopolymers, such as alginate, chitosan, or pectin, to form stable encapsulation matrices, thereby improving the controlled release of vitamins, polyphenols, and essential oils. For example, Kumar et al.^26^ developed pectin-based films containing NADES-curcumin complexes, which exhibited improved UV stability and prolonged antioxidant activity in bakery products.

Despite the growing evidence of efficacy, industrial adoption remains limited by a lack of long-term safety data and the scalability of encapsulation processes.

The physicochemical compatibility of NADES with common encapsulation materials must be systematically assessed, including moisture sensitivity, permeability, and migration potential. Moreover, the absence of a clear regulatory classification for NADES as additives or carriers poses additional hurdles to commercialization.

Future work should focus on combining in vitro digestion models with metabolomic and sensory analyses to evaluate the holistic impact of NADES-based encapsulation systems on food quality and human health.

### 3.7 Animal-Based and Fermented Products

The integration of NADES in animal-based and fermented food systems has garnered significant attention due to their multifunctional properties, which act simultaneously as extraction solvents, antimicrobial agents, and metabolic modulators.

In meat products, NADES-based extracts have demonstrated strong antioxidant and antimicrobial effects. Ghafoor et al.^27^ applied a choline:lactic acid (1:2) NADES extract of rosemary to ground beef and reported a 45% reduction in lipid oxidation and suppression of *Listeria monocytogenes* growth during refrigerated storage. Similarly, Mouratoglou et al.^28^ incorporated oregano extracts obtained using NADES into sausages, maintaining sensory quality and color stability for 10 days, outperforming controls extracted with ethanol.

Beyond their preservation capacity, NADES are also emerging as modulators of fermentation kinetics. Santos et al.^29^ demonstrated that the addition of NADES to mixed-culture fermentations enhanced the formation of short-chain fatty acids while reducing excess ethanol, improving the sensory balance and functional profile of kombucha-type beverages. Mika et al.^30^ further reported that low concentrations of sugar- and acid-based NADES stimulated probiotic growth and increased microbial diversity, suggesting mild prebiotic activity and potential synbiotic applications.

These results collectively highlight NADES as versatile functional ingredients capable of bridging the gap between extraction efficiency and food preservation.

However, most studies remain limited to pilot scale and short storage periods; the long-term microbial ecology and safety implications of NADES–microbiota interactions are still unclear. Future work should quantify their impact on fermentation pathways, metabolite production, and sensory outcomes under industrially relevant conditions.

### 3.8 Edible Packaging and Bioactive Films

NADES have recently been incorporated into biopolymer-based films, such as chitosan, pectin, and starch, as both plasticizers and active carriers for antioxidants and antimicrobials. Their incorporation improves flexibility, barrier properties, and the controlled release of bioactive compounds, enabling multifunctional edible packaging systems. Mika et al.^30^ developed pectin–chitosan composite films containing choline:tartaric acid *N*-acyl-α-d-glucosamine (choline:tartaric acid NADES) and green tea extract, which exhibited enhanced mechanical strength (a 30% increase in tensile strength) and strong antifungal activity against *Aspergillus niger*. Such systems demonstrate the dual function of NADES as structural plasticizers and as vehicles for bioactive retention and release.

NADES reduce oxygen permeability and delay oxidative deterioration, making them suitable for coating perishable foods such as meat, fruits, and dairy products. Zainal-Abidin et al.^8^ reported that films containing choline:glycerol (1:2) maintained antioxidant activity for over 30 days, suggesting controlled diffusion of phenolics through the polymeric matrix.

Despite these advances, migration, sensory neutrality, and consumer safety remain underexplored. Current studies lack standardized protocols to evaluate bioactive release kinetics, film–food interactions, and NADES residual migration into food matrices. Establishing such data is essential for compliance with EFSA and FDA packaging regulations. Taken together, the results discussed in Sections 3.1–3.8 highlight the broad applicability of NADES in food systems, encompassing extraction and stabilization, as well as fermentation, packaging, and preservation.

Table 4 summarizes the most representative applications of NADES reported between 2020 and 2025, while Figure 2 illustrates their relative functional advantages compared with conventional solvents. These results confirm that NADES act as multifunctional enablers of green and efficient food technologies.

**Table 4:** Applications of NADES in Food Systems (2020–2025), Organized by Type of Bioactive Compound^a^

Bioactive compound	NADES system (HBA:HBD)	Food matrix	Application	Yield/Benefit (%)	Reference
Polyphenols	choline:citric acid (1:1)	Pitomba peel	Extraction + incorporation	Greater oxidative stability	Souza et al.^31^
Anthocyanins	choline:fructose (1:1)	Red grape	Extraction + stabilization	88% color preserved (7 days)	Aslan and Doğan^17^
β-Carotene	menthol:caprylic acid (1:1)	Carrot	Lipophilic extraction	+34% vs hexane	Radošević et al.^10^
Catechins	betaine:malic acid (1:2)	Green tea	Encapsulation + digestibility	92% protection (in vitro)	Molnar et al.^2^
Caffeine	choline:urea (1:2)	Coffee husk	Extraction	94% extracted	Zhou et al.^21^
Saponins	choline:glycerol (1:1)	Quinoa	Extraction + bitterness reduction	38% bitterness reduction	Taco et al.^13^
Total polyphenols	choline:acetic acid (1:1)	Stachys byzantina	Extraction + incorporation	Greater color and aroma stability	Silva et al.^12^
Astaxanthin	dodecanol:lauric acid (1:2)	Microalgae	Extraction	25% more than ethanol	Jelić et al.^3^
Isoflavones	choline:sorbitol (1:1)	Oat-based fermented drink	Antioxidant stabilization	46% higher retention postfermentation	Jelić et al.^3^

a Summary of natural deep eutectic solvent (NADES) applications in foods, organized by type of bioactive compound, system used (HBA:HBD), source matrix, type of application, and key comparative results, based on scientific literature published between 2020 and 2025.

**Figure 2:**
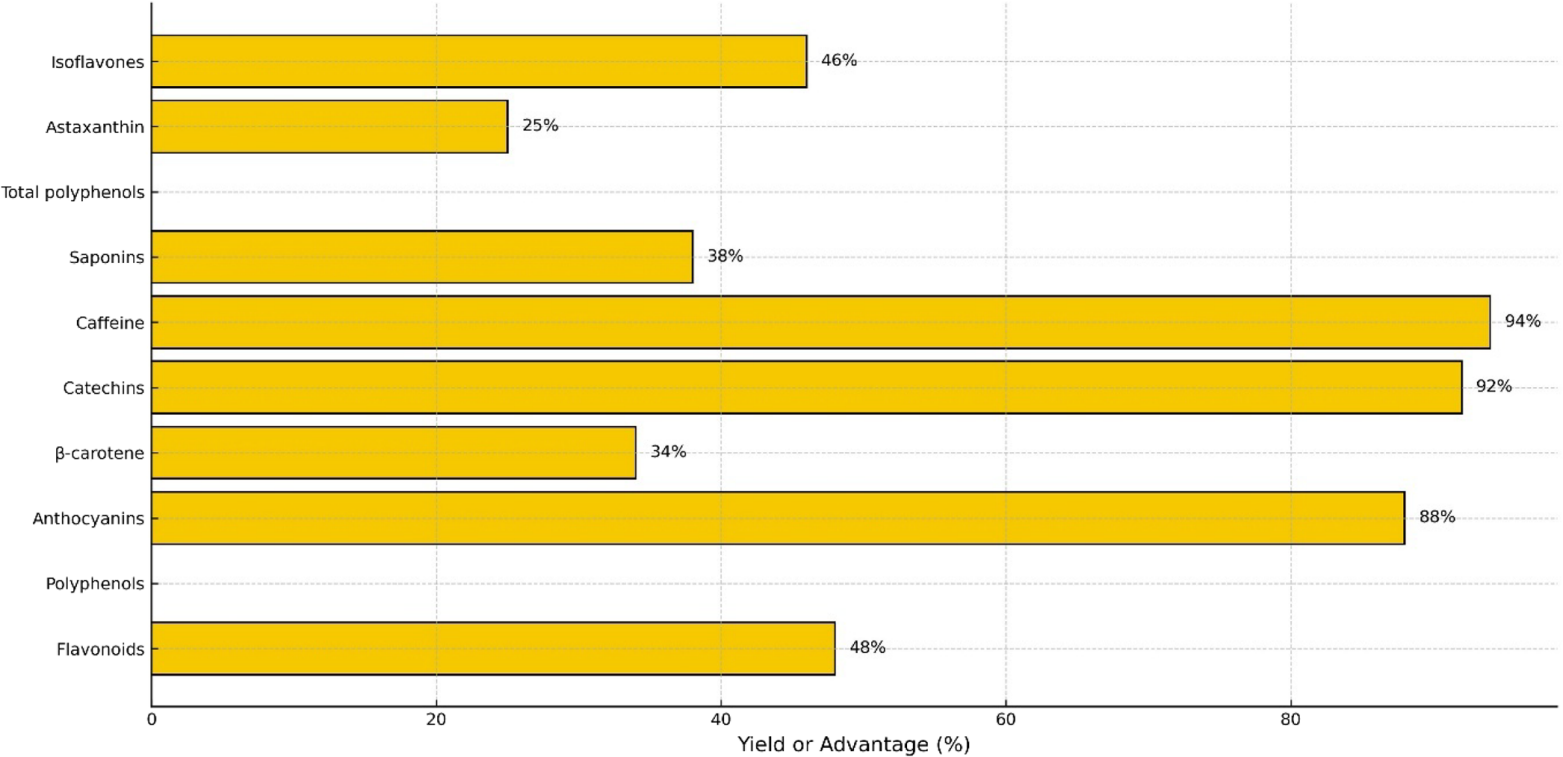
Relative improvement in extraction yield or functional advantage (%) of various bioactive compounds using NADES systems in food applications (2020–2025). Note: Values represent comparative results from representative studies listed in Table 4 rather than averaged data sets.

Figure 2 summarizes the applications of natural deep eutectic solvents (NADES) in foods, organized by type of bioactive compound, solvent system (HBA:HBD), source matrix, type of application, and primary comparative outcomes, according to scientific literature published between 2020 and 2025.

The data presented in Table 4 and Figure 2 reveal clear trends in the efficiency and functionality of NADES across different bioactive compound classes.

Hydrophilic systems based on choline and organic acids (e.g., choline:glycerol, choline:lactic acid, and choline:citric acid) consistently produced higher extraction yields for phenolic compounds, flavonoids, and anthocyanins, typically 30–50% higher than ethanol or methanol controls. Conversely, hydrophobic NADES such as menthol:caprylic acid and dodecanol:lauric acid were more effective for lipophilic pigments like β-carotene and astaxanthin, confirming the strong polarity–solubility relationship described in Section 2.3.2.

Notably, betaine:malic acid systems showed superior performance in encapsulation and digestion stability (≈90% protection of catechins), while choline-based formulations provided better oxidative stability when incorporated into meat or fermented matrices. These comparative outcomes demonstrate the versatility of NADES not only as extraction solvents but also as active carriers within complex food systems.

However, the apparent efficiency gains should be interpreted cautiously, since most studies differ in water content, molar ratios, and extraction conditions.

Standardized benchmarks and comparative data sets are urgently needed to validate the true functional advantages of NADES across different bioactive classes.

### 3.9 Limitations and Regulatory Perspectives

Despite remarkable progress in the design and application of NADES, their translation from laboratory to industry remains limited by regulatory, technological, and methodological barriers.

The primary challenges include:

1. Absence of specific regulatory frameworks from authorities such as the FDA and EFSA, leading to uncertainty regarding classification (solvent, additive, or carrier) and acceptable limits in foods.
2. Lack of standardized toxicological protocols, particularly for complex ternary or quaternary NADES mixtures.
3. High viscosity and mass-transfer limitations, which hinder continuous or large-scale extraction processes.
4. Insufficient sensory and migration studies, delaying market approval and consumer acceptance.

Nevertheless, the scientific and technological outlook is highly promising.

Recent trends indicate the development of biocompatible, food-grade, and responsive NADES formulations that can adapt to environmental stimuli, including pH, temperature, and enzymatic activity.^2^ The integration of NADES into food biorefinery models aligns with sustainability goals by enabling the simultaneous extraction, stabilization, and valorization of agro-industrial byproducts.

## 4 Challenges, Gaps, and Future Perspectives in the Use of NADES in Foods

Despite the increasing volume of research and the promising applications of NADES in the food industry, several critical barriers remain to be addressed before their widespread industrial adoption can be realized. This section provides an overview of the key scientific and technical limitations, identifies current research gaps, and outlines future perspectives based on advances reported between 2020 and 2025.

### 4.1 Technological Barriers and Practical Limitations

From a processing and engineering perspective, the large-scale implementation of NADES in food is still constrained by several technological bottlenecks, particularly those related to viscosity, mass transfer limitations, energy demand, and solvent recovery. In contrast to low-viscosity ethanol–water mixtures, many NADES exhibit viscosities in the range of 100–10,000 mPa·s at room temperature, which markedly increases the resistance to diffusion and hinders efficient mixing, pumping, and filtration.^3,8^ As a result, unit operations that are readily scalable with conventional solvents, such as percolation, counter-current extraction, or continuous stirred-tank systems, become more complex and energy-intensive when NADES are used as the main solvent phase.

High viscosity is frequently mitigated by heating, dilution with water, or the use of cosolvents; however, these strategies introduce further trade-offs. Increasing temperature can accelerate mass transfer but may promote thermal degradation of thermolabile bioactives, while excessive dilution (>40–50% water) can disrupt the eutectic network, gradually shifting the system toward a conventional aqueous solution and reducing the unique solvation properties of NADES.^11,17^ In addition, changes in viscosity and polarity during processing complicate process control and scale-up, as equipment must accommodate broader ranges of rheological behavior than those typically observed for standard food-grade solvents.

Downstream separation and solvent recovery represent another critical technological challenge. Due to the strong hydrogen bonding and specific interactions between NADES and extracted compounds, the separation of bioactives, residual solids, and solvent is often more challenging than in ethanol–water systems, leading to incomplete recovery and the accumulation of impurities.^14,24^ Classical separation techniques, such as liquid–liquid extraction, distillation, and evaporation, are not always directly applicable, especially for nonvolatile NADES, and may require the integration of alternative approaches, including membrane processes, adsorption, antisolvent precipitation, or chromatography. These additional steps increase process complexity, capital expenditure, and operating costs, thereby challenging the economic feasibility of NADES-based operations at an industrial scale.^10,11^

The compatibility of NADES with existing food-processing equipment also remains insufficiently explored. Many pilot-scale studies have relied on batch reactors, laboratory sonicators, or small pressurized cells, which do not accurately reflect the hydrodynamics, shear profiles, or residence-time distributions encountered in industrial extractors, mixers, or heat exchangers. Fouling, corrosion, and changes in material integrity resulting from repeated exposure to highly concentrated organic acids or polyols have rarely been systematically evaluated.^8^ Moreover, the integration of NADES with downstream operations, such as spray drying, freeze-drying, encapsulation, or fermentation, still lacks standardized protocols, which complicates their incorporation into existing production lines.

Finally, there is a scarcity of techno-economic and life-cycle assessment (LCA) studies that quantify whether the environmental and sustainability benefits of NADES offset their higher processing complexity. While NADES are often presented as “green” solvents due to their biodegradability and derivation from biobased components, the total environmental impact depends on solvent synthesis routes, energy requirements for mixing and heating, solvent losses during processing, and the efficiency of recovery and reuse.^2,11^ Without robust comparative data against established ethanol–water or supercritical CO_2_ processes, it remains difficult to define clear decision criteria for when NADES are truly advantageous from a technological and sustainability standpoint. Addressing these technological barriers in parallel with toxicological, regulatory, and sensory evaluations will be essential to move NADES-based processes from laboratory demonstrations to reliable, large-scale food applications.

### 4.2 Toxicological and Regulatory Challenges

The safety evaluation of NADES remains one of the most underexplored areas in food research. Although the individual components of most formulations, such as choline chloride, lactic acid, glycerol, and citric acid, are recognized as GRAS by the U.S. Food and Drug Administration (FDA), their combination into complex eutectic systems may generate physicochemical behaviors that differ from those of the isolated components.

Radošević et al.^10^ reported cytotoxicity values (IC_50_) between 5 and 10% v/v for choline-based NADES in Caco-2 and HepG2 cells, while citric acid–glycerol systems exhibited no detectable toxicity up to 20% v/v, suggesting an acceptable safety margin for food-related applications. Similarly, Molnar et al.^2^ demonstrated that betaine-malic acid and choline-lactic acid mixtures preserved cell viability at concentrations above 85% relevant to extraction and fortification processes.

However, toxicological outcomes are strongly dependent on water content, molar ratio, and hydrogen-bond strength. For instance, formulations with high acidity or high viscosity may induce osmotic stress or membrane disruption at elevated concentrations. Only a limited number of in vivo studies have been conducted to date, with most focusing on short-term exposure.

Therefore, chronic toxicity, bioaccumulation, and metabolic degradation pathways remain poorly characterized and must be addressed before regulatory approval can be achieved.

Although many NADES components are GRAS, the regulation of NADES as combined systems has not yet been established by the FDA/EFSA. The lack of a precise legal classification hinders market entry (additive vs processing aid), residue limits, and labeling. Progress requires harmonized toxicological protocols (including NOAELs), migration studies, and purity criteria specific to NADES mixtures, not only to their individual components.^1,2,9^

### 4.3 Sensory and Consumer Acceptability Gaps

Despite advances in the chemical and microbiological characterization of foods treated with NADES, the sensory acceptability of the resulting products remains poorly documented. Silva et al.^12^ reported good acceptance of fresh sausages enriched with plant extracts obtained using NADES. However, similar studies are lacking for other matrices such as cheeses, sauces, fermented beverages, or plant-based products. Furthermore, the sensory impact of NADES themselves (even at low residual concentrations) is still unclear, especially those containing amino acids, sugars, or organic acids, which may alter taste, odor, or texture. This issue becomes more critical when NADES are added directly as functional ingredients.

Integrated protocols combining hedonic scaling, triangle tests, descriptive profiling, and volatile analysis (GC–MS/e-nose) are needed to quantify thresholds, masking/enhancement effects, and shelf life impacts across demographics.

### 4.4 Scale-Up and Industrial Application Gaps

To date, most applications of NADES remain confined to laboratory or pilot-scale studies. There is a notable scarcity of reports evaluating their performance in continuous industrial production lines, particularly under conditions involving high temperatures, freezing, drying, or homogenization.

Additional bottlenecks include the limited integration of NADES with emerging technologies such as 3D food printing, controlled-release encapsulation systems, and active edible packaging. Although these areas exhibit significant potential for NADES, practical validation is still lacking.^30^

Furthermore, the large-scale production of food-grade NADES presents considerable technical challenges. These include the need for high-purity reagent sourcing and strict control over parameters such as moisture content, stability, and mixture uniformity to ensure consistent batch-to-batch reproducibility. Economic assessments (CapEx/OpEx, solvent recycling logistics) and LCA comparisons versus ethanol/water baselines are also scarce and should be prioritized.

Another critical challenge for industrial implementation is the need to use large volumes of NADES as primary solvents in extraction, fractionation, or formulation steps and the difficulty of reusing these systems over multiple processing cycles. Due to their high viscosity and strong solute–solvent interactions, the complete recovery of NADES from complex food matrices is often impractical, leading to a gradual loss of solvent, accumulation of impurities, and an increased demand for fresh reagents.^9^ In many studies, the reported reuse is limited to only a few cycles with partial retention of performance, indicating that robust regeneration strategies (e.g., membrane separation, antisolvent-assisted recovery, or integration with distillation or adsorption stages) are still required to make NADES-based processes economically and environmentally viable at scale.^8,11^

Table 5 provides an integrated overview of the key gaps and challenges identified in recent literature, highlighting the interconnection between technical, toxicological, and consumer-related factors.

**Table 5:** Main Gaps and Challenges in the Use of NADES in Food Applications by Thematic Area (2020–2025)

Thematic Area	Identified Challenges or Gaps	References
Technical/Processing	High viscosity, limited compound separation, low bioactive recovery efficiency, and interference in chemical reactivity	Zainal-Abidin et al.;^8^ Jelić et al.^3^
Toxicological	Lack of in vivo studies; scarcity of standardized data on chronic toxicity and cytotoxicity of complex mixtures	Suresh et al.;^9^ Ran et al.;^1^ Molnar et al.^2^
Regulatory	Absence of specific international regulations for NADES as food additives; GRAS status limited to individual components	Ran et al.;^1^ Silva et al.;^12^ Jovičić et al.^14^
Sensory/Application	Lack of sensory testing; absence of shelf life data; limited studies on effects on taste, texture, and aroma	Silva et al.;^12^ Aslan and Doğan^17^
Formulation Development	Prevalence of conventional binary NADES; need for developing systems responsive to pH, enzymes, and temperature	Jovičić et al.;^14^ Molnar et al.;^2^ Radošević et al.^10^
Scale-up	Lack of technical and economic feasibility studies for large-scale applications, especially in continuous systems	Radošević et al.;^10^ Bi et al.^24^
Functionality and New Applications	Emerging applications such as encapsulation, biorefineries, and controlled release are still incipient in complex systems	Molnar et al.;^2^ Souza et al.;^22^ Suresh et al.^9^
Consumer Acceptance and Perception	Few data on consumer acceptance regarding labeling, perception of safety, and naturalness	Xie et al.;^32^ Ran et al.;^1^ Zhou et al.^21^
Biocompatibility and Biodegradability	Need to prove postuse residue biodegradability and compatibility with sensitive bioactive compounds	Zainal-Abidin et al.;^8^ Jelić et al.;^3^ Souza et al.^22^

The data summarized in Table 5 reveal that the most persistent challenges in NADES applications are cross-disciplinary and interdependent. Technological and formulation-related issues, such as high viscosity and limited compound separation, are closely linked to toxicological and regulatory gaps, as solvent recovery efficiency and purity directly impact safety profiles. The prevalence of binary NADES systems also indicates that the design of more complex, responsive eutectics remains an underdeveloped research front.^2^

Another pattern emerging from the literature is the imbalance between laboratory success and industrial validation. More than 80% of publications reviewed from 2020 to 2025 report extraction yields and antioxidant performance at bench scale; however, fewer than 10% address scale-up or cost efficiency.^10,11^ This gap highlights the need for techno-economic analyses and life-cycle assessments (LCA) to verify the environmental advantages often attributed to NADES.

From a consumer and sensory perspective, the scarcity of systematic testing indicates that product acceptance could become a bottleneck even if regulatory approval is achieved. Studies such as those by Silva et al.^12^ and Souza et al.^22^ show promising results for meat products; however, similar data for plant-based or dairy matrices are almost nonexistent.

Together, these findings underscore that technological optimization, toxicological validation, and consumer perception must progress in tandem for NADES to become commercially viable in food systems.

### 4.5 Future Perspectives and Innovation Pathways

Despite the current challenges, the future outlook for NADES remains highly promising. In the coming years, several key developments are anticipated:

- An increase in food safety assessments and the achievement of international regulatory approvals.

- The design of smart NADES capable of responding to stimuli such as pH, light, and digestive enzymes.
- Enhanced integration with clean and emerging technologies, including precision fermentation, 3D food printing, and vertical farming.
- Advances in the application of NADES as carriers for bioactive compounds in encapsulation systems and active packaging materials.
- The establishment of publicly accessible databases containing physicochemical and functional data on validated food-grade NADES formulations. In addition, coordinated guidance on labeling and permissible limits would accelerate adoption while preserving consumer trust.

Successful adoption of NADES in food systems will depend on bridging technological optimization, toxicological safety, sensory validation, and regulatory harmonization. A multidisciplinary approach, linking green chemistry, food engineering, toxicology, and consumer science, will be essential to move NADES from laboratory innovations to industrially viable, safe, and sustainable solutions.

### 4.6 Overall Implications

This review critically and comprehensively examines the application of natural deep eutectic solvents (NADES) in the food industry, with a focus on research published between 2020 and 2025. The analysis reveals a growing and significant body of literature dedicated to the development and implementation of NADES as sustainable alternatives to conventional organic solvents, particularly in the extraction, stabilization, and incorporation of bioactive compounds into food matrices. Their relevance spans diverse areas, including plant-derived extracts, agro-industrial byproducts, and animal-based food systems.

The findings indicate that NADES possess highly versatile physicochemical properties—such as the ability to solubilize a broad spectrum of polar and nonpolar compounds, confer antioxidant protection, and demonstrate biodegradability—while showing strong compatibility with emerging extraction techniques like ultrasound-assisted extraction (UAE), microwave-assisted extraction (MAE), and pressurized liquid extraction (PLE). Notably, studies by Radošević et al.^10^ and Da Silva et al.^23^ report superior extraction yields and enhanced preservation of bioactivity when NADES are used in place of traditional solvents such as ethanol, methanol, and hexane.

Beyond extraction, NADES have also shown promise in various functional applications. These include their direct incorporation into meat products,^12^ stabilization of anthocyanins and flavonoids in plant-based foods,^17^ and use in encapsulation systems for controlled release.^2^ However, most current investigations remain limited to laboratory-scale experiments, employing relatively simple binary NADES formulations and focusing predominantly on extractive performance.

Despite notable advancements, several challenges continue to hinder the broader adoption of NADES. The absence of standardized toxicity assessment protocols, combined with the lack of specific regulatory guidelines from international agencies such as EFSA and FDA, remains a significant barrier to commercial use.^1,9^ Additional issues include the high viscosity of certain formulations, difficulties in solute separation postextraction, and limited data regarding sensory impact and shelf life in real food systems.^8,10,11^ These gaps underscore the urgent need for comprehensive in vivo studies, pilot-scale validations, and economic feasibility analyses.

Table 5 of this review consolidates the key technological, regulatory, toxicological, and sensory limitations currently facing the field, outlining priority areas for future research. Moreover, the development of stimuli-responsive NADES, capable of responding to environmental triggers such as pH, temperature, or enzymatic activity, remains underexplored.^2^ Their potential roles in biorefineries, clean label product development, and innovation in plant-based foods also warrant deeper investigation.

A distinguishing feature of this work is its integration of real-world data, recent case studies, and original insights from the authors’ own research. The review organizes NADES applications according to compound classes (e.g., phenolics, carotenoids, saponins), presents updated comparative performance metrics, and introduces an original mapping of current challenges and future opportunities in the field.

NADES represent a highly promising technological platform for sustainable innovation in food science. However, their effective integration into the food industry will require coordinated efforts among researchers, regulatory agencies, and manufacturers. Future investigations must extend beyond extractive efficiency to emphasize safety, functionality, consumer acceptance, and compatibility with real-world processing conditions. The successful transition from potential to practical application will depend on the design of tailored NADES formulations, regulatory clarity, and validation through industrial-scale studies.^2,9,22^
